# Interaction of neuropsychiatric symptoms with *APOE* ε4 and conversion to dementia in MCI patients in a Memory Clinic

**DOI:** 10.1038/s41598-020-77023-z

**Published:** 2020-11-18

**Authors:** Valero Sergi, Marquié Marta, Rojas De Itziar, Espinosa Ana, Moreno-Grau Sonia, Orellana Adelina, Montrreal Laura, Hernández Isabel, Mauleón Ana, Rosende-Roca Maitée, Alegret Montse, Pérez-Cordón Alba, Ortega Gemma, Roberto Natalia, Sanabria Angela, Abdelnour Carla, Gil Silvia, Tartari Juan Pablo, Vargas Liliana, Esteban-De Antonio Ester, Benaque Alba, Tárraga Lluís, Boada Mercè, Ruíz Agustín

**Affiliations:** 1 Research Center and Memory Clinic, Fundació ACE Institut Català de Neurociències Aplicades - Universitat Internacional de Catalunya (UIC), Gran Via Carles III, 85 bis., 08028 Barcelona, Spain; 2 Networking Research Center on Neurodegenerative Diseases (CIBERNED), Instituto de Salud Carlos III, Madrid, Spain

**Keywords:** Neuroscience, Psychology

## Abstract

To date, very few studies have been focused on the impact of the convergence of neuropsychiatric symptoms (NPS) and *APOE* ε4 on the conversion to dementia in patients with Mild Cognitive Impairment patients (MCI), and none has been based in a clinical setting. The objective of the study is to determine the predictive value of additive and multiplicative interactions of NPS and APOE ε4 status on the prediction of incident dementia among MCI patients monitored in a Memory Clinic. 1512 patients (aged 60 and older) with prevalent MCI were followed for a mean of 2 years. Neuropsychiatric symptoms were assessed at baseline using the Neuropsychiatric Inventory Questionnaire. Cox proportional hazards models were calculated. Additive interactions for depression, apathy, anxiety, agitation, appetite, or irritability and a positive ε4 carrier status were obtained, significantly increasing the hazard ratios of incident dementia (HR range 1.3–2.03). Synergistic interactions between NPS and APOE ε4 are identified among MCI patients when predicting incident dementia. The combination of the behavioral status and the genetic trait could be considered a useful strategy to identify the most vulnerable MCI patients to dementia conversion in a Memory Clinic.

## Introduction

﻿Mild Cognitive Impairment (MCI) is a relevant syndromic entity characterized by early stages of cognitive decline with preserved autonomy and it is frequently associated with neurodegenerative diseases, Alzheimer’s disease (AD) being the most prevalent one^1^. As MCI is considered an intermediated diagnosis between normal cognition and dementia, much effort has been dedicated to the identification of those individuals with MCI with a higher vulnerability of conversion to dementia. The study of the simultaneous effect of neuropsychiatric symptoms and genes is increasing its interest in recent years^2^. Considering that a susceptibility condition can be better explained by the confluence of a behavioral status and the effect of one or more genes, the main idea is to highlight the relevance of gene–behavioral interactions. To date, only two studies have looked into the interaction between NPS and *APOE* when determining an increased risk of the hazard of an eventual case of dementia^3,4^. These studies have shown significant interactions between behavioral disturbances and *APOE* ε4 in predicting of incident dementia in cognitively healthy individuals or MCI patients extracted from population-based cohorts. However, nothing is known about the combined contribution of these two risk factors on the conversion to dementia in MCI patients diagnosed and followed in a Memory Clinic. The objective of the present study is to determine the effect of interaction of NPS and *APOE* ε4 on conversion to dementia in a large sample of MCI patients, controlling for several relevant clinical factors.

## Methods

### Participants

Patients were recruited and assessed at the Memory Clinic from Fundació ACE, Institut Català de Neurociències Aplicades (Barcelona, Spain)^5^, from 2005 to 2018. Informed consent was obtained from all participants. The referral center ethics committee (Hospital Clínic i Provincial of Barcelona) approved the patient recruitment and collection protocols were in accordance with ethical standards according to the World Medical Association Declaration of Helsinki—Ethical Principles for Medical Research Involving Human Subjects. All diagnoses were assigned at a daily consensus conference among neurologists, neuropsychologists and social workers. At the time of enrollment patients fulfilled Petersen’s MCI diagnostic criteria^6^, including subjective memory complaints, normal general cognition, preserved performance of daily living activities, absence of dementia, and a measurable impairment in one or more cognitive functions. Patients also had the following characteristics: a Clinical Dementia Rating Scale (CDR)^7^ of 0.5, age older than 60 years of age, functionally literate, and without severe auditory or visual abnormalities including glaucoma and cataracts. Participants received standardized neurobehavioral exams, including neurological examination, neuropsychological testing, and social work evaluations. Information about vascular risk factors (including hypertension, hypercholesterolemia, diabetes mellitus, history of stroke, heart disease) and family history of dementia was provided by the patients or their caregivers. All subjects underwent the neuropsychological battery of Fundació ACE (NBACE)^8^ for diagnostic purposes. A total of 4173 MCI patients had one or more clinical follow-up visits at the Memory Clinic. Among these patients, 2030 had available DNA sample and, among them, 1512 had a basal assessment of NPS. Participants finally included in the study (n = 1512), in comparison with those excluded (n = 2661), were statistically homogenous in age and gender. They had, however, lower MMSE (25.86 vs 26.1; p = 0.002) and more years of education (6.88 vs 6.37; p < 0.001). Both differences showed an effect size (d) < 0.13.

### Measurement of neuropsychiatric symptoms

Neuropsychiatric symptoms were assessed using the Neuropsychiatric Inventory-Questionnaire (NPI-Q). The NPI-Q is a shorter version of the Neuropsychiatric Inventory, a validated clinical instrument^9^. In this study, the Spanish version of the test was used^10^. The NPI is a frequently used measure that assesses 12 NPS (i.e., agitation/aggression, delusion, hallucination, depression/dysphoria, anxiety, euphoria/elation, apathy, disinhibition, irritability/lability, aberrant motor behavior, sleep, and eating/appetite). NPI-Q was filled by the neurologist/geriatrician during the clinical visit according to information provided by the family member/caregiver. The study was focused on the presence or absence of each explored symptom.

### Neuropsychological assessment

All MCI patients completed the neuropsychological battery of Fundació ACE (NBACE)^11^. This diagnostic procedure assesses eight cognitive domains, as follows: (1) Orientation—temporal, spatial, and personal orientation; (2) Attention and working memory—digit spans (forwards and backwards) subtests from the Wechsler adult intelligence scale—third edition (WAIS-III); (3) Processing speed and Executive function—the automatic inhibition subtest from the Syndrome Kurz Test (SKT); phonetic verbal fluency (words beginning with ‘P’ in 1 min); semantic verbal fluency (‘animals’ in 1 min); the similarities subtest from WAIS-III (abbreviated to the first 10 items); (4) Language—repetition (two words and two sentences); verbal comprehension (to correctly execute two simple, two semi-complex, and two complex commands extracted from the Alzheimer’s disease assessment scale (ADAS) and the Barcelona test battery); an abbreviated 15-item Boston naming test; (5) Verbal Learning and Memory—word list learning test from the Wechsler memory scale—third edition (WMS-III) (without using the interference list); (6) Praxis—block design subtests from WAIS-III (abbreviated so that items 6 to 9 were scored only for accuracy (1 point) without a time bonus); imitation praxis (four items); ideomotor praxis (four items); (7) Visual gnosis—the Poppelreuter test, Luria’s clock test, and the 15-objects test; and (8) Global cognition—the Spanish version of the clock test.

### APOE genotyping

Genomic DNA was extracted from peripheral blood using the commercially kit available Chemagic system (Perkin Elmer). The APOE genotypes were determined with the use of fluorogenic allele-specific oligonucleotide probes^12^ (TaqMan assay; Life Technologies, Spain) for rs7412 (Test ID: C_904973_10) and rs429358 (Test ID: C_3084793_20). For the TaqMan assays, PCR and real-time fluorescence measurements were carried out in QuantStudio3 real-time PCR system (Thermo Fisher Scientific, Spain) using the TaqMan Universal Master Mix (ref: 4364341, Life Technologies, Spain) methodology according to manufacturer’s instructions. Polymerase chain reaction was performed as follows: first, a pre-read step for 30 s at 60 °C, a denaturation for 10 min at 95 °C, followed by 40 cycles at 95 °C for 15 s and 60 °C for 1 min, and a post-read stage for 30 s at 60 °C. The genotype was determined using the Genotyping App for Thermo Fisher Cloud by clustering analysis. The laboratory technicians were blinded to other study variables.

### MCI converter and non-converter criteria

Subjects who converted to dementia (including AD^13^ vascular dementia^14^, mixed dementia (AD with cerebrovascular disease), frontotemporal dementia^15,16^, or dementia with Lewy bodies^17^) over the study period, were classified as MCI converters. All of these subjects had a CDR^7^ of 1. In contrast, those subjects who remained stable during follow-ups were classified as MCI non-converters.

### Memory impairment and etiology patterns

MCI subjects were classified as amnestic or non-amnestic, according to Petersen’s criteria^6^. Potential causative factors were also clinically examined to attribute etiology. As multiple causative factors can be recognized in a patient, only the primary was assigned according to its salience. Four MCI patterns were finally generated: degenerative, vascular, psychiatric, and others^18–21^. Patients were classified under the *Other* condition when neither of the aforementioned three causal conditions was identified as preeminent.

### Personal and family history

Family medical history of neurological conditions, including dementia, Huntington’s disease, Parkinson’s disease, psychiatric conditions or Down syndrome were recorded for the MCI participants and encoded as 1 or 0 (present/absent), according to information reported by patients and caregivers. The exploration of these conditions included current or past conditions of parents, grandparents, siblings and children. In order to have a simple approach to all these variables as an adjusting factor, they were combined using Component Analysis. The five conditions were included in the factorial analysis and a solution of one-factor was forced. The resulting factorial loadings were used here as a proxy of the Family medical history of neurological conditions. This new standardized variable was identified in this study as *FamMedHist_comp*. Comorbidities of MCI subjects were also explored. Hypertension, dyslipidemia, diabetes, cardiopathy, stroke, traumatic brain injury, epilepsy, depression, COPD, kidney disease, thyroid disease, osteoarthritis, current or past comorbidities of patients, were also encoded as present or absent (1/0). These 12 variables were processed by a Component Analysis, in the same way as the *FamMedHist_comp* variable. Factorial loadings here obtained were called *MedComorb_comp*.

### Antidepressant and anxiolytic medication

The use of antidepressant and anxiolytic medications was registered. Every variable was coded as 1 if the medication prescription was observed at least once during the follow-up or 0 when no medication was prescribed.

### Analytical procedure

To explore the association between NPS and conversion to dementia, a Chi-Square test was conducted. Hazard ratios (HRs) and 95% confidence intervals (95% CIs) were used to assess the association between the independent variables, NPS and *APOE* genotype, and the outcome of conversion to dementia using Cox proportional hazard models. Interaction effects between NPS and the *APOE* ε4 genotype were the main targets of the analyses. The corresponding and necessary main effects of interactions (the neuropsychiatric symptom and *APOE* effects alone) were also included in the model. Additive and multiplicative interactions were explored. Strengthening the Reporting of Observational Studies in Epidemiology (STROBE) recommendations for the analysis of interactions were followed; that is, we reported different effects of the two risk factors and their joint effect using one reference category, thus providing enough information to calculate interaction on an additive and multiplicative scale^22^. Interactions and main effects were coded following Andersson^23^. Cox models were dually calculated, adjusted or not by age, gender, years of education, baseline MMSE, memory pattern (amnestic vs. nonamnestic), *FamMedHist_comp, MedComorb_comp*, etiology (degenerative, vascular, psychiatric, and others) and prescription of antidepressant and anxiolytic medication. Etiology, as a categorical variable, was recoded by obtaining dummy variables, considering degenerative status as the reference category. In the description of data, and to provide more accurate estimations, especially for NPS with small samples, confidence intervals for means and proportions were calculated by bootstrapping (k = 500). Statistical testing was done at a conventional two-tailed at a level of p < 0.05. Statistical analyses were performed using STATA 15 (Stata Corporation, College Station, Texas).

## Results

At baseline, the prevalence of NPS in the MCI cohort ranged from 55% for depression and anxiety to less than 0.5% for elation/euphoria or motor disturbances. Heterozygous or homozygous *APOE* ε4 genotypes were observed in 31% of the total sample. The follow-up had a mean of 2.03 years (DE = 1.68; median = 1.38; percentile 25 = 0.98; percentile 75 = 2.42). 67.9% of the total sample were amnestic MCI. The suspected etiology for the MCI syndrome was as follows: 31.6% degenerative, 26.7% vascular, 33.5% psychiatric, and 8.2% others. Antidepressant and anxiolytic medication use was observed in 31.2% and 20% of cases, respectively. Conversion to dementia occurred in 58% of patients. Details of demographic and clinical variables, stratified by NPS, are summarized in Table 1. Elation/euphoria and motor disturbances were excluded of analyses because of their low numbers.

**Table 1:** Demographic and clinical characteristics of participants for the total sample and stratified by neuropsychiatric symptoms.

	Total (N = 1512)	Depression/Dysphoria (n = 832)	Apathy (n = 670)	Anxiety (n = 825)	Agitation/Aggression (n = 58)	Night behaviors (n = 432)	Appetite/Eating (n = 123)	Disinhibition (n = 50)	Irritability/Lability (n = 544)	Delusions (n = 52)	Hallucinations (n = 20)
*APOE* ε4^b^	31.02 (28.8–33.4)	31.13 (28.3–34.5)	31.04 (27.5–34.5)	34.4 (30.8–37.7)	31.58 (19.4–44.7)	30 (25.9–34)	32.23 (23.8–41.1)	22.9 (10.9–36.9)	31.73 (28–35.5)	28 (15.9–41.3)	27.8 (9.6–50)
Sex (women)^b^	63.82 (61.3–66.3)	68.6 (65.4–71.8)	58.81 (55.4–62.8)	68.29 (64.9–71.5)	25.44 (13.4–38.1)	66.98 (62.5–71.2)	76.03 (68.3–83.9	47.92 (34.9–60.9)	53.14 (49.3–57.4)	76 (63.2–87.4)	66.67 (41.4–87.5)
Age (years)^a^	76.44/6.71 (76.1–76.8)	76.59/6.79 (76.1–77.1)	76.64/6.62 (76.2–77.1)	76.27/6.78 (75.8–76.8)	76.74/7.46 (74.9–78.7)	76.78/7.11 (76.1–77.4)	77.50/6.86 (76.4–78.7)	77.61/6.17 (76–79.4)	75.96/6.81 (75.4–76.5)	79.51/6.11 (77.8–81.1)	78.49/6.72 (75.1–81.4)
Years of education^b^	6.73/4.16 (6.5–6.9)	6.41/3.96 (6.2–6.7)	6.78/4.14 (3.9–4.4)	6.47/3.99 (6.2–6.8)	7.02/4.33 (5.9–8.3)	6.5/4.08 (6.1–6.9)	6.23/3.77 (5.6–6.9)	7.13/4.65 (5.7–8.3)	6.65/4.18 (6.2–7)	5.26/4.4 (4.1–6.6)	6.5/4.9 (4.4–8.8)
FamMedHist_comp^a^	0/1 (− 0.05–0.05)	− 0.02/0.93 (− 0.07–0.05)	0.06/1.06 (-0.02-0.15)	− 0.00/0.92 (-0.06-0.07)	0.43/1.64 (0.05-0.84)	0.06/1.06 (-0.04-0.16)	0.04/0.87 (-0.1-0.2)	0.43 (0.06–83)	0.08/1.1 (-0.00-0.19)	− 0.21/1 (− 0.51–0.05)	0.37/1.59 (− 0.29–1.22)
MedComorb_comp^a^	0/1 (0.06–0.06)	0.13/0.99 (− 0.07–0.05)	0.15/0.99 (0.07–0.23)	0.06/0.99 (− 0.01–0.13)	− 0.01/1.04 (− 0.28–0.29)	0.16/0.99 (0.07-0.25)	0.24/1 (0.06-0.42)	0.29/0.91 (0.04-0.57)	0.09/1.02 (0.00-0.18)	0.12/1.07 (− 0.2–0.47)	0.38/0.84 (− 0.03–0.75)
MMSE^a^	25.58/2.95 (25.4–25.7)	25.57/2.93 (25.4–25.8)	25.31/2.89 (25.1–25.5)	25.56/2.94 (25.4–25.7)	25.96/2.76 (25.3–26.8)	25.72/2.87 (25.5–26)	25.64/2.64 (25.2–26.1)	25.52/3.19 (24.5–26.4)	25.58/2.91 (25.3–25.8)	24.76/3.38 (23.8–25.8)	25.67/3.14 (24.1–27)
MCI subtype (amnestic)^b^	67.86 (65.4–70.3)	67.2 (63.9–70.2)	69.1 (65.7–72.6)	67.8 (64.7–71.3)	45.61 (31.6–60)	65.36 (60.8–70)	65.29 (57–73.2)	54.17 (40.5–67.5)	66.97 (62.7–70.8)	66 (53.3–79)	55.56 (35.4–78.3)
Years in study^a^	2.04/1.69 (2–2.1)	2.05/1.72 (1.9–2.2)	1.98/1.66 (1.8–2.1)	2.15/1.84 (2–2.3)	2.16/1.68 (1.7–2.6)	2.06/1.82 (1.9–2.2)	1.92/1.66 (1.6–2.2)	1.71/1.59 (1.3–2.2)	2.19/1.83 (2–2.3)	1.79/1.39 (1.4–2.2)	2.8/3.12 (1.6–4.7)
**Etiology**											
Degenerative	31.5 (29–33.8)	24 (21.2–27.1)	29.1 (25.9–32.5)	25.6 (22.8–28.4)	49.1 (36.2–64)	24.7 (20.1–28.7)	19.8 (26.1–43)	50 (37.2–64.6)	32.3 (28.1–35.9)	42 (28.9–57.1)	44.4 (22.2–69.8)
Vascular	26.7 (24.5–29.1)	26.4 (23.4–29.7)	29.4 (25.9–33.1)	24.8 (21.8–27.6)	15.8 (6.9–25.7)	24.7 (20.7–28.6)	32.3 (23.7–40.9)	29.2 (17–41.5)	28.6 (24.7–32.5)	26 (14.6–37)	22.2 (5.1–44.7)
Psychiatric	33.5 (30.9–36)	44.1 (40.5–47.5)	35.7 (32.2–39)	43.4 (40.1–46.6)	26.3 (14.6–38.6)	44.4 (29.7–49.3)	46.3 (38–55.1)	14.6 (4.2–24.5)	32.5 (28.4–36.6)	26 (13.9–38.4)	27.8 (8.3–50)
Others	8.2 (6.9–9.6)	5.4 (3.8–6.8)	5.8 (4.3–7.8)	6.2 (4.4–7.9)	8.8 (1.9–16.5)	6.3 (4.1–8.7)	1.7 (0–4.5)	6.3 (0–14.3)	6.6 (4.7–8.7)	6 (0–13.6)	5.6 (0–21.4)
**Medication**											
Antidepressant	31.3 (29.1–33.5)	31.7 (28.4–35.1)	30.4 (27.2–34.3)	33.5 (30.8–36.5)	36.8 (24.8–48.4)	30.9 (26.5–35.3)	27.3 (19.9–34.9)	22.9 (11.3–35.5)	32.5 (28.4–36.5)	30 (18–43.7)	33.3 (10.9–57.1)
Anxiolytic	20 (18.2–22)	21.2 (18.5–23.9)	20 (16.9–22.9)	21.5 (18.8–24.5)	26.3 (14.3–38.1)	18.8 (15.3–22.8)	15.7 (9.1–22-6)	8.3 (0–16.8)	19.6 (16.5–23.2)	20 (9.8–32)	16.7 (0–36.4)
Conversion to dementia^b^	58.2 (55.7–60.8)	58.5 (54.4–61.2)	63.1 (59.8–66.8)	58.69 (55.4–61.5)	70.2 (57.6–82.3)	51.63 (46.8–56.2)	67.77 (59.5–76)	64.58 (51.2–77.2)	60.33 (56.5–64.6)	76 (63.3–88.3)	61.11 (38.7–85.3)

^a^Means/standard deviations with bootstrapped confidence interval 95% (k = 500).

^b^Percentages with bootstrapped confidence interval 95% (k = 500).

When exploring the distribution of NPS between *APOE* ε4 carriers and noncarriers (Table 2), only anxiety symptoms presented a significant association with this allele (p = 0.002). In particular, results showed that among *APOE* ε4 carriers, the proportion of anxiety was lower than among noncarriers. For the rest of NPS, the proportion of *APOE* ε4 carriers was statistically homogeneous.

**Table 2:** Neuropsychiatric symptoms in APOE4 carriers and noncarriers.

	*APOE* ε4 carrier (%)	*APOE* ε4 Noncarrier (%)	Chi test	p
Depression	30.88	31.13	0.02	0.918
Apathy	31	31.04	0	0.984
Anxiety	27	34.39	9.57	0.002
Agitation/aggression	31	31.58	0.01	0.926
Night behaviors	31.42	30	0.29	0.589
Appetite/eating	30.91	32.23	0.09	0.764
Disinhibition	31.28	22.92	1.52	0.218
Irritability/lability	30.62	31.73	0.2	0.652
Delusions	31.12	28	0.22	0.639
Hallucinations	31.06	27.78		1^a^

^*a*^Fisher exact test.

Clinical differences between converters and non-converters are presented in Table 3. Older age, female sex, fewer years of education, *APOE* ε4 carrier status, lower baseline MMSE scores were more common among converters (p < 0.008 for all variables). Amnestic MCI patients were statistically more prevalent among converters than non-converters (p < 0.001). Concerning etiology, the degenerative condition appeared heterogeneously distributed between converters and non-converters. Close to 38% of converters were characterized by this condition, while within non-converters this percentage was 22.9%. Vascular and psychiatric etiological conditions were also comparable between both groups. The “Other” condition was more prevalent among non-converters.

**Table 3:** Clinical overview between converters and nonconverters.

	Nonconverters (n = 632)	Converters (n = 880)	T or Chi statistics	p value
Age	74.4 (6.71)^b^	77.91 (6.32)^b^	10.38	< 0.001
Female	59.8^c^	66.7^c^	7.28	0.007
Education (years)	7.23 (3.99)^b^	6.38 (4.24)^b^	3.99	< 0.001
*APOE* ε4 carrier	25^c^	35.3^c^	17.9	< 0.001
MMSE	26.44 (2.57)^b^	24.96 (3.05)^b^	10.19	< 0.001
FamMedHist_comp	0.04 (1.1)^b^	− 0.03 (0.92)^b^	1.37	0.159
MedComorb_comp	-0.03 (1.03)^b^	0.02 (0.98)^b^	1.09	0.275
Amnestic MCI subtype	62.7^c^	71.6^c^	13.05	< 0.001
**Etiology**			52.99	< 0.001
Degenerative	22.9^c^	37.7^c^		
Vascular	27.1^c^	26.5^c^		
Psychiatric	37.8^c^	30.5^c^		
Others	12.5^c^	5.3^c^		
**NPS symptoms**				
Depression	55.2^c^	54.9^c^	0.01	0.939
Apathy	39.1^c^	48.1^c^	11.68	< 0.001
Anxiety	53.8^c^	54.9^c^	0.14	0.714
Agitation/aggression	2.7^c^	4.5^c^	2.99	0.083
Night behaviors	32.9^c^	25.2^c^	10.3	0.001
Appetite/eating	6.2^c^	9.3^c^	4.53	0.033
Disinhibition	2.7^c^	3.5^c^	0.581	0.446
Irritability/lability	34^c^	37.2 ^c^	1.44	0.23
Delusions	1.9^c^	4.3 ^c^	5.99	0.014
Hallucinations	1.1^c^	1.3 ^c^	0.01	0.991

^*a*^Chi test statistics are shown for categorical variables, t-test for quantitative variables.

Regarding NPS, the presence of baseline apathy, agitation/aggression, night behaviors, appetite/eating, and disinhibition showed a significant effect on conversion to dementia. Night behaviors showed an inverse profile (higher prevalence of this NPS in the non-converter group). Depression and anxiety were the most prevalent symptoms in the total sample, but along with irritability, delusions, and hallucinations showed a nonsignificant differential effect on conversion to dementia.

**Figure 1:**
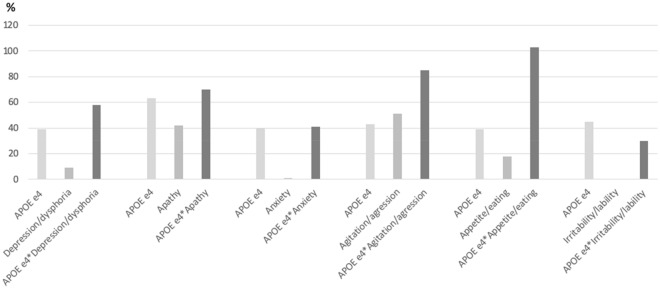
Incremental risk (%) of conversion associated to significant additive interactions and the corresponding main effects of *APOE* ε4 and NPS. The incremental risk is calculated considering patients *APOE* ε4 noncarriers and without NPS symptoms as the reference condition.

Table 4 shows Cox regression analyses for NPI, *APOE* ε4, and its interaction on survival time to conversion to dementia. When non-adjusted results were explored, additive interaction of *APOE* ε4 with depression, apathy, anxiety, appetite, and night behaviors emerged as significant. In a multiplicative scale, none of the interactions showed a significant effect. Under adjusting effect, all significant results previously reported for additive interactions, except for night behaviors, emerged again as significant. Multiplicative interaction for apathy was the only one with a significant result but showing an inverse effect (HR = 0.74). Appetite and agitation presented the highest incremental risk of conversion to dementia (> 85%) in the additive interactions with APOE ɛ4 (Fig. 1).

**Table 4:** *APOE* ε4 and NPS main effects and their interaction in additive and multiplicative scale.

	HR(CI95%)^a^	p value^a^	Additive interaction p value^a^	Multiplicative interaction p value^a^	HR(CI95%)^b^	p value^b^	Additive interaction p value^b^	Multiplicative interaction p value^b^
*APOEε4* Depression/dysphoria APOEε4*depression	1.53 (1.24–0.88)	< 0.001			1.39 (1.13–1.72)	0.002		
	1 (0.85–1.18)	0.964			1.09 (0.92–1.29)	0.335		
	1.42 (1.17–1.72)		0.001		1.58 (1.29–1.94)		< 0.001	
	0.92 (0.7–1.22)			0.563	1.04 (0.78–1.38)			0.769
*APOEε4* Apathy APOEε4*Apathy	1.59 (1.32–1.93)	< 0.001			1.63 (1.34–1.98)	< 0.001		
	1.31 (1.11–1.54)	0.001			1.42 (1.19–1.69)	< 0.001		
	1.72 (1.42–2-11)		< 0.001		1.71 (1.39–2.09)		< 0.001	
	0.82 (0.63–1.09)			0.177	0.74 (0.55–0.98)			0.038
*APOEε4* Anxiety APOEε4*anxiety	1.48 (1.2–1.83)	< 0.001			1.4 (1.13–1.74)	0.002		
	0.88 (0.74–1.03)	0.121			1.01 (0.85–1.2)	0.888		
	1.3 (1.08–1.56)		0.006		1.45 (1.19–1.76)		< 0.001	
	1 (0.75–1.32)			0.985	1.02 (0.77–1.36)			0.881
*APOEε4* Agitation/aggression APOEε4*Agitation/aggression	1.47 (1.27–1.69)	< 0.001			1.43 (1.23–1.65)	< 0.001		
	1.14 (0.77–1.7)	0.503			1.51 (1.01–2.26)	0.043		
	1.61 (0.95–2.74)		0.076		1.85 (1.07–3.19)		0.027	
	0.96 (0.5–1.87)			0.912	0.85 (0.44–1.69)			0.652
*APOEε4* Night Behaviors APOEε4*Night behaviors	1.39 (1.18–1.63)	< 0.001			1.39 (1.18–1.64)	< 0.001		
	0.8 (0.66-0.97)	0.021			0.81 (0.67–0.98)	0.032		
	1.34 (1.06–1.71)		0.015		1.23 (0.96–1.57)		0.101	
	1.22 (0.88–1.66)			0.237	1.08 (0.79–1.51)			0.611
*APOEε4* Appetite/eating APOEε4*Appetite/eating	1.44 (1.24–1.67)	< 0.001			1.39 (1.2–1.62)	< 0.001		
	1.22 (0.92–1.63)	0.174			1.18 (0.87–1.57)	0.275		
	2.04 (1.42–2.94)		< 0.001		2.03 (1.41–2.93)		< 0.001	
	1.16 (0.73–1.87)			0.533	1.24 (0.77–1.99)			0.369
*APOEε4* Disinhibition APOEε4* Disinhibition	1.46 (1.26–1.67)	< 0.001			1.43 (1.23–1.65)	0.268		
	1.37 (0.90–2.07)	0.142			1.26 (0.83–1.93)	< 0.001		
	2.97 (1.48–5.98)		0.002		1.93 (0.95–3.90)		0.070	
	1.49 (0.66–3.37)			0.339	1.06 (0.47–2.41)			0.886
*APOEε4* Irritability/lability APOEε4*Irritability/lability	1.43 (1.20–1.71)	< 0.001			1.45 (1.21–1.73)	< 0.001		
	0.91 (0.77–1.08)	0.307			0.94 (0.79–1.12)	0.503		
	1.38 (1.12–1.7)		0.003		1.3 (1.05–1.62)		0.015	
	1.05 (0.79–1.39)			0.76	0.96 (0.71–1.28)			0.756
*APOEε4* Delusions APOEε4*delusions	1.49 (1.30–1.72)	< 0.001			1.45 (1.25–1.67)	< 0.001		
	1.85 (1.27–2.69)	0.001			1.41 (0.97–2.06)	0.072		
	1.52 (0.79–2.95)		. 207		1.37 (0.70–2.67)		0.356	
	0.55 (0.25–1.18)			0.125	0.67 (0.31–1.46)			0.311
*APOEε4* Hallucinations APOEε4*hallucinations	1,48 (0.54–2.05)	< 0.001			1.44 (1.25–1.67))	< 0.001		
	1.05 (1.29–1.70	0.884			0.94 (0.47–1.87)	0.869		
	0.54 (0.13–2.16)		0.384		0.43 (− 11–1.74)		0.237	
	0.35 (0.07–1.62)			0.178	0.31 (0.07–1.51)			0.148

^a^Nonadjusted.

^b^Adjusted by sex, age, years of education, MMSE, FamMedHist_comp, MedComorb_comp, amnestic and etiology MCI subtype, and antidepressant/anxiolytic medication.

When considering significant additive interactions under the adjusted model, those associated with agitation/aggression and appetite/eating showed a specific profile. In contrast with depression/dysphoria, apathy or anxiety, interaction effects for agitation/aggression presented the highest risk between all NPS and the most discriminant effects in comparison to the corresponding behavioral and genetic main effects. Irritability had a unique effect within this set of results: its additive interaction was (statistically) lower than the sum of the corresponding main effects.

Within the MCI converters group, it was observed that 74% participants converted to AD, 12% to vascular dementia and 4.3% to Frontotemporal dementia, while the rest converted to different etiologies of dementia. When differentiating between AD vs other forms of dementia, a logistic regression analysis was performed, including the ten relevant neuropsychiatric symptoms as predictors, and adjusted by the same variables used in the survival analysis. Apathy emerged as the only significant symptom when detecting conversion to other kind of dementia different than AD, although with a weak effect (Wald = 4.20, p = 0.041, 95%CI: 1.01–1.96).

## Discussion

﻿Although relevant differences can be observed among studies, behavioral disturbances have been considered a prevalent condition among MCI patients^24^ and have been associated with a significant and increased risk of incident dementia, particularly AD^25^. At the same time, these symptoms have been considered some of the earliest clinical manifestations of prodromal AD^26^.

The study results have to be highlighted for two reasons. First, this is the first one exploring the connection between behavioral and genetic risk factors for conversion to dementia in MCI patients in a Memory Clinic. Second, our results evidence the relevance of exploring the incremental predictive value of interaction between risk factors beyond standard approaches based on the analyses of only risk factors’ main effects. Our data demonstrate a synergistic effect of depression, apathy, anxiety, agitation, appetite and irritability, and a positive ε4 carrier status. These significant results are particularly relevant because there is no previous evidence, according to meta-analytical approaches, that the *APOE* ɛ4 genotype and behavioral symptoms are associated in MCI patients, except in the case of anxiety, where a differential distribution of the symptom seems to be associated to the *APOE *ε4 condition^2^. Interestingly, all these observations are concordant with our study’s conclusions. The most relevant consideration, however, is that a higher prevalence of *APOE* ε4 carriers is not expected when a NPS is present, at least for the majority of symptoms. Behavioral disturbances and *APOE* ε4 are independent factors that, when they converge in MCI patients, manifest themselves most severely predicting conversion to dementia. Interestingly, nearly all NPS involved in additive interactions in the present study had been previously identified as the most remarkable ones when differentiating between cognitively normal adults and AD patients^27^.

Our results are convergent with those observed in a population-based study by Burke et al.^3^ where, in a sample of more than 11,000 cognitively intact participants, delusions, hallucinations, agitation, depression, anxiety, elation apathy, disinhibition, irritability, motor disturbances, appetite, and sleep disturbances appeared to have additive interactions with *APOE* ε4 as risk factors for dementia conversion. In a study by Pink et al.^4^, which examined 332 MCI patients from a population-based sample, additive interactions with APOE ɛ4 were also observed for apathy and depression. Hence, we propose that previous observations are generalizable to the MCI phenotype and might be of interest as predictors of the conversion to dementia in this population.

A multiplicative interaction with a significant outcome is an exceptional result. Only in Burke et al.^3^ a remarkable interaction in this scale was observed for delusions and motor disturbances. In our study, apathy was the only behavioral condition with a significant result in a multiplicative interaction with APOE ɛ4, but its effect has to be interpreted in terms of a reduction in the risk rate. Exceptional or poor results associated with multiplicative interactions in the context of Cox regression models, however, should not be surprising: it means that the observed combined effect is larger (or smaller) than the product of the individual effects while an additive interaction is observed when its combined effect is larger (or smaller) than the sum of the two exposures. The additive scale has been identified by several epidemiologists, in fact, as the appropriate strategy to assess interaction because it provides more applicable explanations for biological events than multiplicative interaction^28,29^.

Several relevant clinical variables were included as adjusting factors in the present study. This approach is suitable because it tries to control the heterogeneous clinical profile that MCI patients present in a Memory Clinic. Participants were characterized not only in terms of standard demographic variables but also in personal medical comorbidity and family history of medical diseases, memory, etiological conditions, and antidepressant and anxiolytic medication prescriptions. Comparable synergistic results with *APOE* were observed for depression, anxiety, apathy, appetite, or irritability, either under the influence of adjustment variables or without them. These convergent results could be interpreted in terms of estimations’ relevance: the synergistic connection between the behavioral condition and *APOE* seems to be scarcely modulated by these other relevant clinical variables. Of course, the present study has not explored the effect of other variables. However, according to the clinical relevance of those included, and the magnitude and direction of results, the convergent effect of NPS and *APOE* ε4 seem to be highly consistent and, given the lack of other evidence, the results could be interpreted in terms of an intrinsic association between the combined effect of both risk factors and conversion to dementia in MCI patients.

When focusing on the detail of the hazard ratio sizes observed in the additive interactions between NPS and APOE ɛ4, it is observed that synergistic effects have an incremental risk of dementia conversion ranging from 30% to more than 100% compared to nonaffected MCI peers. The average risk is close to 60%. This estimation is particularly relevant when observing that the incremental mean risk of conversion to dementia associated with *APOE* ε4 and NPS’ main effects are 44% and 14%, respectively. Agitation/aggression and appetite appeared as the most discriminant conditions for dementia conversion, not for the symptoms themselves (appetite has a low predictive value compared with other NPS), but when presented simultaneously with the genetic trait. This specific risk profile, where the additive interaction emerges as a high predictive condition in contrast to its main effects, has to be highlighted. MCI patients affected by agitation/aggression or appetite/eating symptoms are not so frequent, but when they additionally present an *APOE* ε4 carrier status become the patients with the poorest prognosis, that is, have the highest risk of progression to dementia.

Knowing the biological mechanisms that underlie is, of course, a relevant scientific target by itself but is also a key objective when trying to identify better treatments. Knowing these mechanisms may help us define new neurochemical mechanisms and better understand how pharmacologic and non-pharmacologic treatments help affected people. Unfortunately, the current understanding of neuropsychiatric symptoms' neurobiology is limited, especially when considering preclinical and prodromal stages of AD^31^. Scientific literature has considered that different patterns of NPS are associated with distinct MCI subtypes, and differences in conversion rates between NPS could be partially consequence of these clinical subtypes. For example, more severe agitation/hyperactivity symptoms over time are observed in MCI patients with anamnestic profile subtype^30^. Other explanations have been formulated connecting the occurrence of particular NPS with specific brain networks or circuits in the brain^32^ or proposing that brain changes, presented in preclinical stages, could be explaining the differential clinical progression of patients, including NPS manifestations^33^. When focusing on genetic research, one central idea is to suggest that genes identified as risk factors of developing AD could also be risk factors for developing particular NPS. Some studies have shown consistent associations between APOE ɛ4 and psychosis in AD. Heritability of psychotic symptoms in AD is estimated to be up to 61%^34^, and, in fact, it has been postulated that AD with psychosis could be considered a specific phenotype with a genetic basis^35^. However, in cognitively impaired subjects, and as it was previously reported, associations between NPS and APOE ɛ4 are unclear^2^. Beyond APOE, although it has been demonstrated that that sortilin-related receptor 1 gene (SORL1) and the ATP-binding cassette, subfamily A, member 7 gene (ABCA7) are associated with AD, the connection of these high-risk genes with NPS occurrence has to be also considered inconclusive^36^. NPS in AD and its preclinical and prodromal conditions have a heterogeneous presentation. They are changeable and do not constitute a unitary or homogeneous status. This natural manifestation of the symptoms could be explaining, at least partially, the difficulties in finding consistent results.

The limitations of the study have to be stated as well. The first one is the limited extension of follow-ups. Data up to only two years after the initial clinical evaluation have been explored. The exploration of MCI as a prodromal stage of dementia should be studied with longer follow-ups to determine the consistency of estimated synergistic hazard ratios. A second consideration is associated with the universe of NPS that were analyzed. Elation/euphoria and motor disturbances were excluded from analyses because of their low prevalence, despite the analysis of a cohort of more than 1500 patients. The solution to this selective loss of information may have a problematic approach in the context of a Memory Clinic. First, these symptoms are rare among MCI patients. Large samples of patients are needed to consistently approach an interaction analysis, something that is usually difficult in clinical settings. Second, even in the context of a Memory Clinic that works under an integrated care paradigm^5^, patients with these particular behavioral conditions are sometimes referred to other more specialized healthcare resources, losing the opportunity of a conversion estimation. Finally, it is well known that APOE ε4 has been identified as a risk factor for AD, while in this study, the conversion rates are calculated for all forms of dementia. Under our consideration, this potential loss of precision when using APOE status in an interaction with a NPS has a relative relevance. First because, as reported above, in the converted subgroup of MCI, the majority of cases are AD (three out of 4 cases) and, second, because the main idea was to identify critical variables when predicting a conversion, any kind of conversion, in the context of an applied setting.

## Conclusions

This study identifies, in a Memory Clinic setting, additive interactions between depression, apathy, anxiety, agitation, appetite, and irritability and *APOE* ε4 as predictors of conversion to dementia in MCI patients. The neuropsychiatric state and the genetic trait are essentially independent factors between them, but when they converge in a patient, the combination emerges as the most discriminant condition for conversion to dementia, beyond the impact of most of the single risk factors. Results of this study represent a step further in the identification of the most exposed MCI patients to dementia conversion and, for the first time, in an applied setting. To date, the *APOE* status cannot be treated, but the neuropsychiatric condition of MCI patients could be targeted by direct or indirect interventions, pharmacological or not. Professionals working in clinical settings can take advantage of these results, not only trying to mitigate the intensity and frequency of current behavioral disturbances but also improving the quality of life of affected people.

## Data Availability

Data is available on request to the main author.

## References

[1] Hanfelt JJ, Peng L, Goldstein FC, Lah J (2018). Latent classes of mild cognitive impairment are associated with clinical outcomes and neuropathology: analysis of data from the National Alzheimer’s Coordinating Center. Neurobiol. Dis..

[2] Banning LCP, Ramakers IHGB, Deckers FRJ, Aalten P (2019). Apolipoprotein E and affective symptoms in mild cognitive impairment and Alzheimer’s disease dementia: a systematic review and meta-analysis. Neurosci. Biobehav. Rev..

[3] Burke SL, Maramaldi P, Cadet T, Kukull W (2016). Neuropsychiatric symptoms and Apolipoprotein E: associations with eventual Alzheimer’s disease development. Arch. Gerontol. Geriatr..

[4] Pink A (2015). Neuropsychiatric symptoms, APOE 4, and the risk of incident dementia: A population-based study. Neurology..

[5] Boada M (2014). Design of a comprehensive Alzheimer’s disease clinic and research center in Spain to meet critical patient and family needs. Alzheimers Dement..

[6] Petersen RC (2004). Mild cognitive impairment as a diagnostic entity. J. Intern. Med..

[7] Morris JC (1993). The clinical dementia rating (CDR): current version and scoring rules. Neurology..

[8] Alegret M (2012). Normative data of a brief neuropsychological battery for Spanish individuals older than 49. J. Clin. Exp. Neuropsychol..

[9] Kaufer DI (2000). Validation of the NPI-Q, a brief clinical form of the neuropsychiatric inventory. J. Neuropsychiatry Clin. Neurosci..

[10] Boada M (2002). Neuropsychiatric Inventory Questionnaire (NPI-Q): validación española de una forma abreviada del Neuropsychiatric Inventory (NPI). Neurología..

[11] Alegret M (2013). Cut-off scores of a brief neuropsychological battery (NBACE) for Spanish individual adults older than 44 years old. PLoS ONE.

[12] Livak KJ (1999). Allelic discrimination using fluorogenic probes and the 5’ nuclease assay. Genet. Anal. Biomol. Eng..

[13] McKhann GM (2011). The diagnosis of dementia due to Alzheimer’s disease: recommendations from the National Institute on Aging-Alzheimer’s Association workgroups on diagnostic guidelines for Alzheimer’s disease. Alzheimers Dement..

[14] Roman GC (1993). Vascular dementia: diagnostic criteria for research studies. Report of the NINDS-AIREN International Workshop. Neurology..

[15] Neary D (1998). Frontotemporal lobar degeneration: a consensus on clinical diagnostic criteria. Neurology..

[16] Rascovsky K (2011). Sensitivity of revised diagnostic criteria for the behavioural variant of frontotemporal dementia. Brain.

[17] McKeith IG, Boeve BF, Dickson DW (2017). Diagnosis and management of dementia with Lewy bodies. Neurology..

[18] Albert MS (2011). The diagnosis of mild cognitive impairment due to Alzheimer’s disease: recommendations from the National Institute on Aging-Alzheimer’s Association workgroups on diagnostic guidelines for Alzheimer’s disease. Alzheimers Dement..

[19] De Mendonça A, Ribeiro F, Guerreiro M, Garcia C (2004). Frontotemporal mild cognitive impairment. J. Alzheimer Dis..

[20] Nordlund A, Rolstad S, Klang O, Lind K, Hansen S, Wallin A (2007). Cognitive profiles of mild cognitive impairment with and without vascular disease. Neuropsychology..

[21] Sachdev P (2014). Diagnostic criteria for vascular cognitive disorders: a VASCOG statement. Alzheimer Dis Assoc Disord..

[22] von Elm E (2007). The strengthening the reporting of observational studies in epidemiology (STROBE) statement. Epidemiology..

[23] Andersson T, Alfredsson L, Källberg H, Zdravkovic S, Ahlbom A (2005). Calculating measures of biological interaction. Eur. J. Epidemiol..

[24] Apostolova LG, Cummings JL (2008). Neuropsychiatric manifestations in mild cognitive impairment: a systematic review of the literature. Dement. Geriatr. Cogn. Disord..

[25] Rosenberg PB (2013). The association of neuropsychiatric symptoms in MCI with incident dementia and alzheimer disease. Am. J. Geriatr. Psychiatry..

[26] Taragano FE, Allegri RF, Lyketsos C (2008). Mild behavioral impairment: a prodromal stage of dementia. Dement. Neuropsychol..

[27] Steinberg M (2013). Point and 5-year period prevalence of neuropsychiatric symptoms in dementia: the Cache County Study. Int. J. Geriatr. Psychiatry..

[28] Knol MJ (2011). Estimating measures of interaction on an additive scale for preventive exposures. Eur. J. Epidemiol..

[29] Knol MJ, Egger M, Scott P, Geerligs MI, Jan P (2009). When one depends on the other: reporting of interaction in case-control and cohort studies. Epidemiology.

[30] De Vito AN, Calamia M, Weitzner DS, Bernstein JPK (2018). Examining differences in neuropsychiatric symptom factor trajectories in empirically derived mild cognitive impairment subtypes. Int. J. Geriatr. Psychiatry..

[31] Johansson, M., et al. Apathy and anxiety are early markers of Alzheimer’s disease. *Neurobiol. Aging*. Accessed on 19 Oct 2020. 10.1016/j.neurobiolaging.2019.10.008 31735378

[32] Rosenberg PB, Nowrangi MA, Lyketsos CG (2015). Neuropsychiatric symptoms in Alzheimer’s disease: what might be associated brain circuits?. Mol. Aspects Med..

[33] Geda YES (2013). Neuropsychiatric symptoms in Alzheimer’s disease: past progress and anticipation of the future. Alzheimer’s Dement..

[34] Bacanu SA (2005). Heritability of psychosis in Alzheimer disease. Am. J. Geriatr. Psychiatry..

[35] DeMichele-Sweet MA, Sweet RA (2010). Genetics of psychosis in Alzheimer’s disease: a review. J. Alzheimers Dis..

[36] Huang, M.F., *et al*. Genetics of neuropsychiatric symptoms in patients with Alzheimer’s disease: a 1-year follow-up study. Accessed 10 Sept 2020. 10.1111/pcn.13150 32909371

