# Author Correction: Interaction of neuropsychiatric symptoms with *APOE* ε4 and conversion to dementia in MCI patients in a Memory Clinic

**DOI:** 10.1038/s41598-021-84389-1

**Published:** 2021-02-22

**Authors:** Sergi Valero, Marta Marquié, Itziar De Rojas, Ana Espinosa, Sonia Moreno‑Grau, Adelina Orellana, Laura Montrreal, Isabel Hernández, Ana Mauleón, Maiteé Rosende‑Roca, Montse Alegret, Alba Pérez‑Cordón, Gemma Ortega, Natalia Roberto, Angela Sanabria, Carla Abdelnour, Silvia Gil, Juan Pablo Tartari, Liliana Vargas, Ester Esteban‑De Antonio, Alba Benaque, Lluís Tárraga, Mercè Boada, Agustín Ruíz

**Affiliations:** 1 Research Center and Memory Clinic, Fundació ACE Institut Català de Neurociències Aplicades - Universitat Internacional de Catalunya (UIC), Gran Via Carles III, 85 bis., 08028 Barcelona, Spain; 2 Networking Research Center on Neurodegenerative Diseases (CIBERNED), Instituto de Salud Carlos III, Madrid, Spain

Correction to: *Scientific Reports* https://doi.org/10.1038/s41598-020-77023-z, published online 18 November 2020

The original version of this Article contained errors in the spelling of the authors Sergi Valero, Marta Marquié, Itziar De Rojas, Ana Espinosa, Sonia Moreno‑Grau, Adelina Orellana, Laura Montrreal, Isabel Hernández, Ana Mauleón, Maiteé Rosende‑Roca, Montse Alegret, Alba Pérez‑Cordón, Gemma Ortega, Natalia Roberto, Angela Sanabria, Carla Abdelnour, Silvia Gil, Juan Pablo Tartari, Liliana Vargas, Ester Esteban‑De Antonio, Alba Benaque, Lluís Tárraga, Mercè Boada & Agustín Ruíz which were incorrectly given as Valero Sergi, Marquié Marta, Rojas De Itziar, Espinosa Ana, Moreno-Grau Sonia, Orellana Adelina, Montrreal Laura, Hernández Isabel, Mauleón Ana, Rosende-Roca Maitée, Alegret Montse, Pérez-Cordón Alba, Ortega Gemma, Roberto Natalia, Sanabria Angela, Abdelnour Carla, Gil Silvia, Tartari Juan Pablo, Vargas Liliana, Esteban-De Antonio Ester, Benaque Alba, Tárraga Lluís, Boada Mercè & Ruíz Agustín respectively.

These errors have now been corrected in the PDF and HTML versions of the Article.

